# Traumatic laser in situ keratomileusis flap dislocation with epithelial ingrowth, *Propionibacterium acnes* infection, and diffuse lamellar keratitis: A case report

**DOI:** 10.1097/MD.0000000000019257

**Published:** 2020-03-06

**Authors:** Yung-Ching Chang, Yuan-Chieh Lee

**Affiliations:** a Department of Ophthalmology, Buddhist Tzu Chi General Hospital; b Department of Ophthalmology and Visual Science; c Institute of Medical Sciences, Tzu Chi University, Hualien, Taiwan.

**Keywords:** LASIK, flap dislocation, epithelial ingrowth, Propionibacterium acnes, infectious keratitis, diffuse lamellar keratitis, case report

## Abstract

**Rationale:**

Traumatic flap dislocation might occur anytime after laser in situ keratomileusis (LASIK), but it is rarely concomitantly complicated with epithelial ingrowth, infectious keratitis, and diffuse lamellar keratitis altogether. Here we report a case of traumatic LASIK flap inversion with epithelial ingrowth, *Propionibacterium acnes* infection, and diffuse lamellar keratitis.

**Patient concerns:**

A 42-year-old man receiving bilateral LASIK surgery 10 years ago complained of right eye pain for 6 days after twig injury. Temporal flap inversion with epithelial ingrowth and dense infiltration at the interface were noted.

**Diagnoses:**

Traumatic LASIK flap inversion with epithelial ingrowth, Propionibacterium acnes infection and diffuse lamellar keratitis.

**Interventions:**

Removal of corneal epithelium around the flap inversion site, flap lifting, scraping of epithelial ingrowth, removal of the dense infiltrate, alcohol soaking, interface irrigation with antibiotics, and flap reposition were performed. Diffuse lamellar keratitis was noted postoperatively. Culture of the infiltrate revealed *P acnes*. The infiltrate subsided and the cornea cleared up under topical antibiotics and steroid.

**Outcomes:**

The visual acuity returned to 20/20. No recurrent epithelial ingrowth or infiltrate was noted during the follow-up.

**Lessons:**

This is the first report of Propionibacterium acnes keratitis after traumatic flap inversion. Although epithelial ingrowth, infectious keratitis, and diffuse lamellar keratitis all developed after the flap inversion, early recognition and proper intervention lead to a good result without sequels.

## 1 Introduction

The laser in situ keratomileusis (LASIK) procedure for correcting refractive errors consists of the creation of a corneal flap, ablation of the stroma by excimer laser, and reposition of the flap. Creating a corneal flap poses risks to the integrity of the corneal surface. The low-grade wound healing between the corneal flap and the stromal bed maintains corneal transparency, but on the other hand, leads to weak flap adhesion. Flap dislocation might occur any time even as long as 14 years after LASIK.^[1]^ Here we report a case of traumatic flap inversion with epithelial ingrowth, *Propionibacterium acnes* infection, and diffuse lamellar keratitis 10 years after LASIK. Written informed consent for the publication of this case and any additional related information was taken from the patient involved in the study.

## 2 Case report

**Figure 1:**
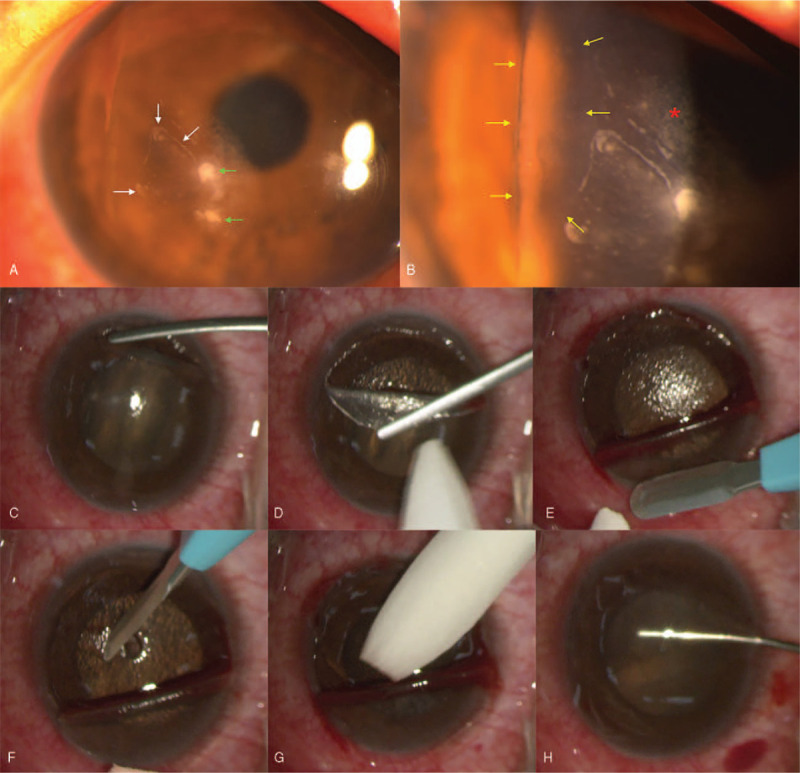
(A) Biomicroscopic examination revealed temporal flap inversion with epithelial ingrowth (white arrows) and 2 dense infiltrations at the interface (green arrows). (B) The border of the inverted flap was marked with yellow arrows. Interstitial keratitis was noted at paracentral cornea (red asterisk). (C–H) The operation procedures were smoothly performed as follows: (C) wide removal of corneal epithelium around the flap inversion site starting from at least 1 mm peripheral to the original LASIK wound; (D) flap lifting and eversion of the inverted flap; (E) scraping of epithelial cells at both stromal bed and under surface of the flap; (F) removal of the 2 dense infiltrates for culture; (G) 70% alcohol soaking for 20 seconds; (H) balanced salt solution irrigation and flap reposition with bandage contact lens application. LASIK = laser in situ keratomileusis.

A 42-year-old man with a history of bilateral myopic LASIK surgery 10 years ago complained of right eye pain for 6 days after injured by a twig. Examination revealed a best corrected visual acuity (BCVA) of 20/100 in the right eye and 20/20 in the left eye. Initial slit lamp examination described 2 suspected ulcers with satellite lesions. Scraping for smear and culture was done, and topical 5% natamycin, 1% voriconazole, and levofloxacin were prescribed, but in vain. The patient was referred to our clinic 3 days later. Temporal flap inversion was noted. There was no epithelial defect at flap inversion site, but epithelial ingrowth, 2 dense infectious infiltrations, and lamellar interstitial keratitis at the interface were demonstrated (Fig. 1A, B). Wide removal of corneal epithelium around the flap inversion site (at least 1 mm peripheral to the original LASIK wound), flap lifting, scraping of ingrowth epithelial cells on both stromal bed and under surface of flap sides, removal of the 2 dense infiltrates for culture, and then 70% alcohol soaking for 20 seconds, interface irrigation with vancomycin (0.4 mg/mL) and voriconazole (0.16 mg/mL), and flap reposition with bandage contact lens were smoothly performed (Fig. 1C–H). Diffuse lamellar keratitis with sands of Sahara pattern was noted on the first postoperative day. Topical 1% Voriconazole, 0.5% Levofloxacin, and 0.1% Betamethasone were given every hour around the clock. Later, the culture of the 2 dense infiltrates revealed *P acnes*, and topical antibiotics were shifted to 0.5% levofloxacin and 3% vancomycin Q2H around the clock at daytime. The cornea cleared up under topical antibiotics and steroids. Topography revealed symmetric smooth surface. The visual acuity returned to 20/20. No recurrent epithelial ingrowth or infiltrate was noted in the following year.

## 3 Discussion

LASIK is a generally safe and predictable procedure for correction of refractive errors. Early flap slippages usually occur within the first day or 2 after surgery^[2]^; while late LASIK flap dislocations are less common but may happen at any time after LASIK due to the diminished wound healing.^[3]^ Late flap dislocations have been reported to be caused by sharp objects such as screwdriver^[4]^ or fingernail,^[5]^ or high-velocity blunt ocular trauma such as basketball^[6]^ or falling pecan.^[7]^ The strength of trauma might be violent as airbag^[7]^ or as mild as self-removal with soft contact lens.^[8]^ The flap dislocation in our case was caused by a twig 10 years after the LASIK procedure.

Epithelial ingrowth into the interface between the flap and stromal bed is a complication of LASIK flap dislocation.^[9]^ Epithelial ingrowth with direct communication and supply from peripheral regenerating epithelium could lead to progressive ingrowth under the flap. A broad area of epithelial ingrowth could hinder the exchange of nutrients and metabolites. Hence a delay in treatment could result in flap melting, increased scarring, and induction of irregular astigmatism.^[1]^ On seeing our patient, there was no epithelial defect stained with fluorescein. Instead, the epithelial cells grow not only into the interface but over the inverted flap. To prevent the continuous supply from the peripheral regenerating epithelium, a wider debridement of the epithelium more peripheral to the original LASIK wound, and those on the bare stroma bed, inverted flap, and both sides of the interface were performed. 70% alcohol to the bed and the undersurface of the flap for 20 seconds and bandage contact lens after reposition of the flap were performed to prevent the recurrence of flap dislocation and epithelial ingrowth.

Infectious keratitis is a potentially sight-threatening interface complication following LASIK. Atypical mycobacteria are most notorious and responsible for some outbreaks.^[10–13]^ Gram-positive cocci are also common etiology in recent years.^[14–16]^ However, these infectious keratitides are mostly introduced intraoperatively. A literature search of infectious keratitis following traumatic flap dislocation revealed etiologies of Nocardia^[17]^ and Enterobacter.^[18]^ In contrast, *P acnes* keratitis following flap dislocation, like that in our patient, has not been reported before. *P acnes* is a relatively slow-growing, anaerobic, gram-positive bacillus. It is often a normal flora of the skin, hair follicles, and conjunctiva.^[19]^*P acnes* is the most frequently isolated bacteria from the conjunctival sac,^[20]^ and may be responsible for about one-tenth of infectious keratitis.^[21,22]^ However, delayed or confused diagnosis on presentation is possible because of its prolonged incubation period and small and deep stromal infiltration.^[22]^*P acnes* infection has once been reported 2 days after presbyopic LASIK,^[23]^ in which a delayed diagnosis was noted. The authors reflected on their treatment and suggested lifting the flap, irrigation, cleaning, and scraping for cultures.^[23]^ Our case, fortunately, was treated with lifting the flap, irrigation with vancomycin, and scraping for culture. The positive culture result was available on day 11, like the average incubation period of *P acnes*.^[22]^

Diffuse lamellar keratitis was described in the late traumatic displacement of LASIK flaps following reposition of flaps on early postoperative days.^[24–26]^ Flap lift and irrigation might be needed in severe cases to prevent flap necrosis or permanent scarring if inflammation worsens.^[16]^ In our patient, diffuse lamellar keratitis developed within the first postoperative day and responded well to aggressive frequent topical steroid use in the early stage.

In conclusion, we reported a traumatic flap dislocation complicated with epithelial ingrowth, *P acne* keratitis and sand of Sahara syndrome. Early recognition, prompt surgical intervention, and sufficient topical treatment could still lead to a good visual outcome.

## Author contributions

**Conceptualization:** Yuan-Chieh Lee.

**Data curation:** Yung-Ching Chang, Yuan-Chieh Lee.

**Formal analysis:** Yung-Ching Chang, Yuan-Chieh Lee.

**Supervision:** Yuan-Chieh Lee.

**Validation:** Yuan-Chieh Lee.

**Writing – original draft:** Yung-Ching Chang.

**Writing – review & editing:** Yuan-Chieh Lee.

Yuan-Chieh Lee orcid: 0000-0002-8486-8549.
